# Color Shift Failure Prediction for Phosphor-Converted White LEDs by Modeling Features of Spectral Power Distribution with a Nonlinear Filter Approach

**DOI:** 10.3390/ma10070819

**Published:** 2017-07-18

**Authors:** Jiajie Fan, Moumouni Guero Mohamed, Cheng Qian, Xuejun Fan, Guoqi Zhang, Michael Pecht

**Affiliations:** 1 College of Mechanical and Electrical Engineering, Hohai University, Changzhou 213022, China; mmguero@yahoo.fr; 2 Changzhou Institute of Technology Research for Solid State Lighting, Changzhou 213161, China; xfan@lamar.edu (X.F.); G.Q.Zhang@tudelft.nl (G.Z.); 3 Beijing Research Center, Delft University of Technology, Delft 2628, The Netherlands; 4 Department of Mechanical Engineering, Lamar University, Beaumont, TX 77710, USA; 5 EEMCS Faculty, Delft University of Technology, Delft 2628, The Netherlands; 6 Center for Advanced Life Cycle Engineering, University of Maryland, College Park, MD 20742, USA; pecht@umd.edu

**Keywords:** LEDs, color shift failure, spectral power distribution, nonlinear filter, reliability and failure analysis

## Abstract

With the expanding application of light-emitting diodes (LEDs), the color quality of white LEDs has attracted much attention in several color-sensitive application fields, such as museum lighting, healthcare lighting and displays. Reliability concerns for white LEDs are changing from the luminous efficiency to color quality. However, most of the current available research on the reliability of LEDs is still focused on luminous flux depreciation rather than color shift failure. The spectral power distribution (SPD), defined as the radiant power distribution emitted by a light source at a range of visible wavelength, contains the most fundamental luminescence mechanisms of a light source. SPD is used as the quantitative inference of an LED’s optical characteristics, including color coordinates that are widely used to represent the color shift process. Thus, to model the color shift failure of white LEDs during aging, this paper first extracts the features of an SPD, representing the characteristics of blue LED chips and phosphors, by multi-peak curve-fitting and modeling them with statistical functions. Then, because the shift processes of extracted features in aged LEDs are always nonlinear, a nonlinear state-space model is then developed to predict the color shift failure time within a self-adaptive particle filter framework. The results show that: (1) the failure mechanisms of LEDs can be identified by analyzing the extracted features of SPD with statistical curve-fitting and (2) the developed method can dynamically and accurately predict the color coordinates, correlated color temperatures (CCTs), and color rendering indexes (CRIs) of phosphor-converted (pc)-white LEDs, and also can estimate the residual color life.

## 1. Introduction

Artificial lighting consumes around 19% of the world’s total energy, which produces approximately 10% of all carbon emitted in the world [1,2]. Light-emitting diode (LED), as one of solid-state lighting (SSL) sources, produces visible light via electroluminescence, which converts electricity to light without relying on heat for radiation. Therefore, LEDs are much more efficient than traditional lighting sources and have become a comparatively low-energy-consuming, long-lasting, and environmentally friendly alternative. In the last few decades, people have been making efforts to improve the quantum efficiency of LED. Tremendous progress has been made for the SSL technology due to the breakthroughs in the development of high-performance LEDs via improvement in extraction efficiency of both LED and organic LED (OLED) [3,4,5,6], and novel photon down converters [7]. This ensures wide adoption of SSL and will cut down the energy usage. However, the mass application of LEDs still faces many barriers, such as high cost, time- and cost-consuming qualification tests, and unreliable lifetime predicted by current methods [8,9].

By using a blue light source converted by phosphors to obtain white light emission, phosphor-converted white LEDs (pc-WLEDs) are becoming one of the alternatives to traditional general lighting sources due to their advantages in energy saving, environment-friendliness, color controllability, and long lifetime. The three failure modes that usually happen in LEDs are catastrophic failure (lighting suddenly turns off), luminous flux degradation, and color shift [8,9]. However, most of the previous studies only used the luminous flux data to calculate the lifetime of LEDs, but it is not the sole characteristic of LEDs. Owing to the expansion of LED applications, the color quality and color consistency of LED light sources has attracted much attention in some color-sensitive application fields, such as museum lighting, healthcare lighting and displays [10]. For this reason, the reliability concern for LEDs is changing from the high luminous efficiency to color quality and color consistence. Meanwhile, the chromaticity (or color) has already been recognized as another indicator of a LED’s “end of life” recommended by the Next Generation Lighting Industry Alliance (NGLIA) of the U.S. Department of Energy (DoE) [11]. However, most current research on the reliability of LED light sources is still focused on luminous flux depreciation [12] rather than on color shift. Moreover, most of the current color shift models are empirical [13] and lack physical meaning, which will definitely impact the accuracy of lifetime prediction.

As the spectral power distribution (SPD), defined as the radiant power distribution emitted by a light source at a range of visible wavelength, contains the most fundamental luminescence mechanisms of an LED, it is usually used as the quantitative inference of an LED’s optical characteristics, including both photometric and colorimetric performances. For example, B. M. Song and B. T. Han used the SPD deconvolution method to evaluate the yellow-to-blue ratio and phosphor power conversion efficiency for pc-WLEDs [14] and analyzed the degradation failure mechanisms on the lumen, correlated color temperature (CCT) and color rendering index (CRI) parameters [15]. Moreover, H. T. Chen and S. Y. Hui developed a tricolor spectral modeling method to dynamically predict the CCTs and CRIs for pc-WLEDs with the photoelectrothermal theory [16]. In addition, M. H. Chang et al., applied the similarity-based metric test to extract the features from SPDs of pc-WLEDs and detect the anomalies for LEDs aged under a degradation test with a k-nearest neighbor-kernel density-based clustering technique [17]. Recently, C. Qian et al. proposed a method to decompose the SPD of LED lamp with the asymmetric Gaussian model and predict the lumen maintenance and color coordinates by estimating the features of the proposed statistical model [18] and also used two asymmetric double sigmoidal (Asym2sig) models, representing the blue light and phosphor converted light peaks respectively, to predict the photometric and colorimetric characteristics of a pc-WLED [19]. The color coordinate shift of LED lamps aged under accelerated ageing tests was investigated by M. Cai et al., [20] and a power model was proposed to predict the color shift process of LED products. However, as reviewed, few of the current studies on SPDs can combine the failure mechanism analysis and residual color lifetime prediction together to assess the reliability of pc-WLEDs during degradation testing.

To investigate the color shift failure mechanisms and predict the residual color lifetime for a pc-WLED aged under a degradation test, this paper proposes a model-based prognostic method by extracting the features of an SPD with the statistical functions with both Gaussian and Lorentzian model firstly, and then modeling the shift trajectories of features of SPDs with a nonlinear filtering approach that delivers a recursive and stochastic parameter estimation by dynamically updating measurements. Finally, with the predicted SPDs, the color coordinates in the color space of Commission Internationale de L’Eclairage (CIE) (CIE 1976), (*u*′,*v*′), the CCT and the CRI will be estimated to qualify the color shift failure of pc-WLED.

The remainder of this paper is organized as follows: In Section 1, the test sample used in this study and its SPDs collected during an accelerated degradation test are given to validate the proposed methods. Section 2 introduces the theory and methodology used in this paper, that includes the luminous mechanisms of pc-WLEDs with an SPD, the feature extraction method for SPDs, and the nonlinear filtering model used for the feature estimation. Section 3 provides validation results and discussions. Finally, the concluding remarks and possible directions for future research are presented in Section 4.

## 2. LED Test Sample and Accelerated Degradation Test

In this section, a high-power pc-WLED package test sample is introduced as the research object of this paper and the accelerated degradation test designed for the selected test sample was implemented for SPD data collection.

**Figure 1:**
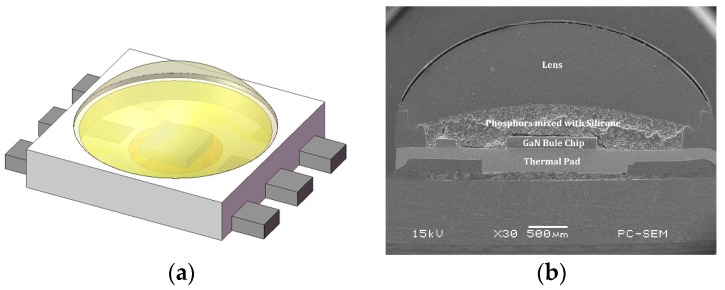
(**a**) The 3D model of selected light-emitting diodes (LED) package; (**b**) its packaging materials and construction shown in the scanning electron microscope image of cross-section.

The LED package used in this study is one type of high-brightness pc-WLEDs from Avago (Type: 3-W high-power WLED light source with the part number as ASMT-JN31-NTV01 [21]), which is manufactured with a GaN blue chip and a monochromatic (yellow) phosphor. The LED package layout and its packaging materials and construction are shown in Figure 1, which indicates that the mechanism for generating white light from the test vehicle is a combination of blue light emitted by a GaN chip and the excited yellow light emission from a phosphor layer.

In this study, an accelerated degradation test was designed for the selected pc-WLED package, which was electrically driven by the DC current (*I*_c_ = 200 mA) provided by a DC power supply (Model: Agilent E3611A). The thermal chamber provided a constant aging temperature (*T*_a_ = 90 °C) for this test. After finishing a 23-h cycle aging, the test sample was removed from the thermal chamber to be cooled to the ambient temperature for SPD data measurement by a Gigahertz-Optik BTS256-LED tester (Türkenfeld, Germany) When the measurement was finished, the test sample was then returned to the thermal chamber to undergo the next round of aging until its color shift failure happened.

**Figure 2:**
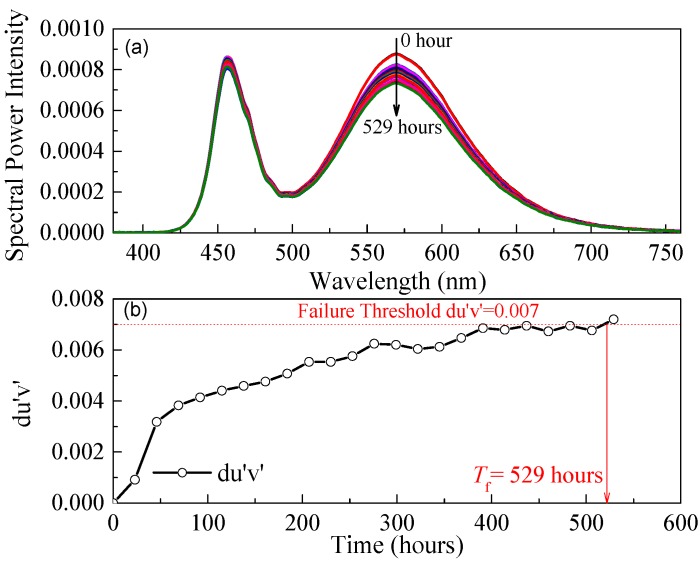
(**a**) Spectral power distribution (SPD) data collected under the accelerated degradation test; (**b**) color shift failure time *T*_f_ = 529 h defined as when *du*′*v*′ = 0.007.

Normally, the Euclidean distance between the original color coordinates and shifted coordinates in the CIE 1976 color space, *du*′*v*′, is used to represent the color shift of LEDs [22]. The International Electrotechnical Commission (IEC) developed a criterion to characterize the color shift failure based on specific color coordinates, which can be defined in terms of the numbers of standard deviations of color matching (SDCM). For general lighting applications, the color shift failure threshold of seven-step SDCM (approximately *du*′*v*′ = 0.007), which is recommended by both the Energy Star Programs of the U.S. Department of Energy [23] and ANSI/NEMA [24], is used in this paper. Figure 2 shows the collected SPD data until the color shift failure happened at the time *T*_f_ = 529 h.

## 3. Theory and Methodology

The three methodologies used in this paper are provided in this section, which include the introduction of luminous mechanisms and calculation of color coordinates for pc-WLEDs with an SPD, the extraction of SPD features with a proposed statistical method, and the estimation of time dependence of the SPD features using a nonlinear filtering based state-space model.

### 3.1. Luminous Mechanisms of pc-WLEDs

**Figure 3:**
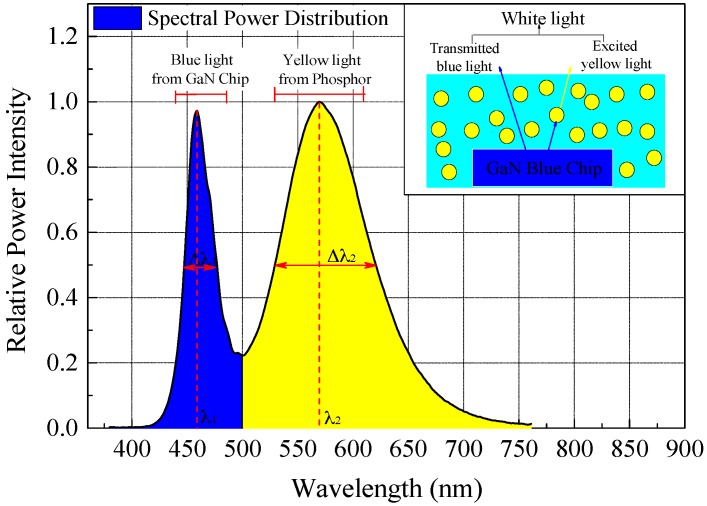
SPD and luminous mechanism of the selected phosphor-converted white LED (pc-WLED) package.

As reviewed in the introduction, because an SPD contains the basic physical information about a light source, it is usually used as the quantitative inferences of optical characteristics (such as luminous flux, color coordinates, correlated color temperature, and color rendering index). The SPD of the selected pc-WLED package and its luminous mechanism are shown in Figure 3. The white light is produced by a combination of the blue light from the GaN chip and the excited yellow light emitted by the yellow phosphor. Therefore, there are two dominant relative power intensity distributions in the SPD spectrum of an LED, which represent the performances of the GaN chip (380~500 nm for blue light) and phosphor (500~780 nm for yellow light), respectively.

In this paper, color coordinates were chosen as the indicator of color shift failure of a pc-WLED and the CCTs and CRIs are also predicted to qualify its color quality. The color coordinates in CIE color space are the basic concepts of colorimetry that quantify and physically describe human color perception [22]. The color coordinates in CIE 1976 color space, (*u*′,*v*′), calculated by Equations (1) and (2), are widely used to represent the chromaticity state of WLEDs, because the color difference is proportional to the geometric difference in this color space. For this reason, they were chosen as the indicator of color shift failure of pc-WLEDs in this paper.

(1) $$u\prime =\frac{4X}{X+15Y+3Z}$$

(2) $$v\prime =\frac{9Y}{X+15Y+3Z}$$

(3) $$X=\int \bar{x}(\lambda )SPD(\lambda )d\lambda$$

(4) $$Y=\int \bar{y}(\lambda )SPD(\lambda )d\lambda$$

(5) $$Z=\int \bar{z}(\lambda )SPD(\lambda )d\lambda$$

where *X*, *Y*, and *Z* are the tristimulus values, which can be obtained by integrating the SPD function, *SPD*(*λ*), with the standard color-matching functions $\bar{x}(\lambda )$, $\bar{y}(\lambda )$, and $\bar{z}(\lambda )$ [15].

### 3.2. SPD Feature Extraction with Statistical Method

**Figure 4:**
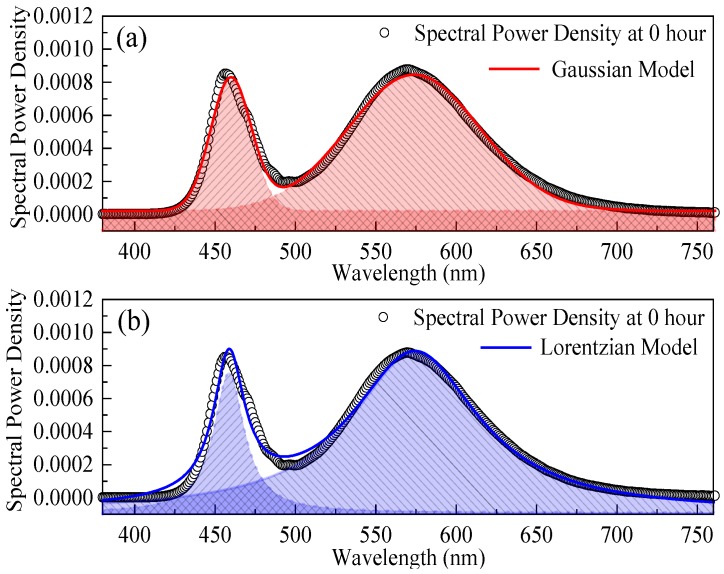
Feature extraction from the initial SPD of pc-WLED package with both Gaussian and Lorentzian models (the red (**a**) and blue (**b**) dash lines with shadow areas represent the Gaussian and Lorentzian models respectively).

As the two emission distributions representing the characteristics of blue LED chips and phosphors of a pc-WLED are similar to the bell-shaped curves, this paper extracted the features of an SPD by multi-peak curve-fitting with statistical functions. In this study, two widely used statistical functions, Gaussian (Equation (6)) and Lorentzian (Equation (7)) models, were used and the curve-fitting results are compared in Figure 4.

Gaussian model:

(6) $$y=y_{0}+\frac{A_{B}}{w_{B}\sqrt{\frac{\pi }{2}}}e^{-2{\left(\frac{x-\lambda_{B}}{w_{B}}\right)}^{2}}+\frac{A_{Y}}{w_{Y}\sqrt{\frac{\pi }{2}}}e^{-2{\left(\frac{x-\lambda_{Y}}{w_{Y}}\right)}^{2}}$$

Lorentzian model:

(7) $$y=y_{0}+\frac{2A_{B}}{\pi }\cdot \frac{\Delta \lambda_{B}}{4{\left(x-\lambda_{B}\right)}^{2}+\Delta {\lambda_{B}}^{2}}+\frac{2A_{Y}}{\pi }\cdot \frac{\Delta \lambda_{Y}}{4{\left(x-\lambda_{Y}\right)}^{2}+\Delta {\lambda_{Y}}^{2}}$$

where *y*_0_ is the baseline offset, *A* is the total area under the curve from baseline, *λ* is the center of the peak, Δ*λ* is the full width of the peak at half height, and *w* equals two standard deviations, that is approximately 0.849 the width of the peak at half height. *B* and *Y* represent LED chip and phosphor, respectively.

As shown in Figure 4, the red and blue solid lines are the fitting results for the entire SPD of the pc-WLEDs measured at initial time. And the features of the GaN LED chip and phosphors can be modeled by the Gaussian and Lorentzian expressions with red and blue dash lines with shadow areas, respectively. The feature extraction results are given in Table 1, in which seven parameters of each statistical model are extracted. As shown, the *R*^2^ values of two models, which are closer to 1, indicate that both statistical functions have well goodness-of-fitting results for the SPD of this type of pc-WLED package. According to the quantum point of view, the recombination in *p*-*n* junction is governed by the electron transition probability to a fundamental state that depends on the coordinated configuration for the electronic/vibrational levels in the blue LED chip. The recombination probability function usually follows a discrete Poisson distribution and it can be assumed as a continuous Gaussian function to describe the SPD of blue LED chip [25]. Otherwise, as compared to the fitting results in the phosphor part, the Lorentzian model is more suitable. Thus, in this paper, both two statistical models were used to extract the features of SPDs collected from the aged test sample.

### 3.3. Color Shift Failure Prediction with Nonlinear Modeling

According to the previous work [13], the color shift of pc-WLEDs is always the nonlinear process during aging. As shown in Equations (1)–(5), in order to predict the color shift failure of a pc-WLED package, it is necessary to track the shift trajectories of features extracted from the collected SPDs during the long-term aging test. Therefore, this paper developed the color shift failure prediction method by modeling the extracted features from two statistical distributions as a function of time with nonlinear approaches.

The particle filter (PF) method has been considered as one of the solutions for state-space estimation in nonlinear and non-Gaussian systems [26]. Previously, the least-squares regression (LSR) method was widely used to conduct a batch-processing estimation by minimizing the sum of the residuals between the actual measurements and the calculated values. The PF method, on the other hand, delivers a recursive and stochastic parameter estimation by dynamically updating measurements and possesses higher prediction accuracy for the nonlinear degraded states [27]. Thus, this paper used the PF method to estimate the extracted features from SPDs by updating the measurement model with the Bayesian dynamic approach. PF always uses a set of particles to approximate the predicted state as a posterior probability density distribution, *x_k_* ~ *p*(*x_k_*|*z*_1:*k*_), with sequential Monte Carlo (SMC) simulation [28]. The state-space model of this study can be expressed as follows [29]:

State model:

(8) $$x_{k}=f(x_{k-1},\alpha_{k})$$

Measurement model:

(9) $$z_{k}=h(x_{k}+\upsilon_{k})\;\upsilon_{k}\;\sim \;N(0,\delta^{2})$$

where *k* is the time (or cycle) step index, *x_k_* is the degradation (or shift) state, *α_k_* is the model parameter, *z_k_* is the measurement data, *υ_k_* is the measurement noise, and *ʘ_k_*(*x_k_*, *α_k_*, *δ_k_*) is the vector of parameters in PF.

**Figure 5:**
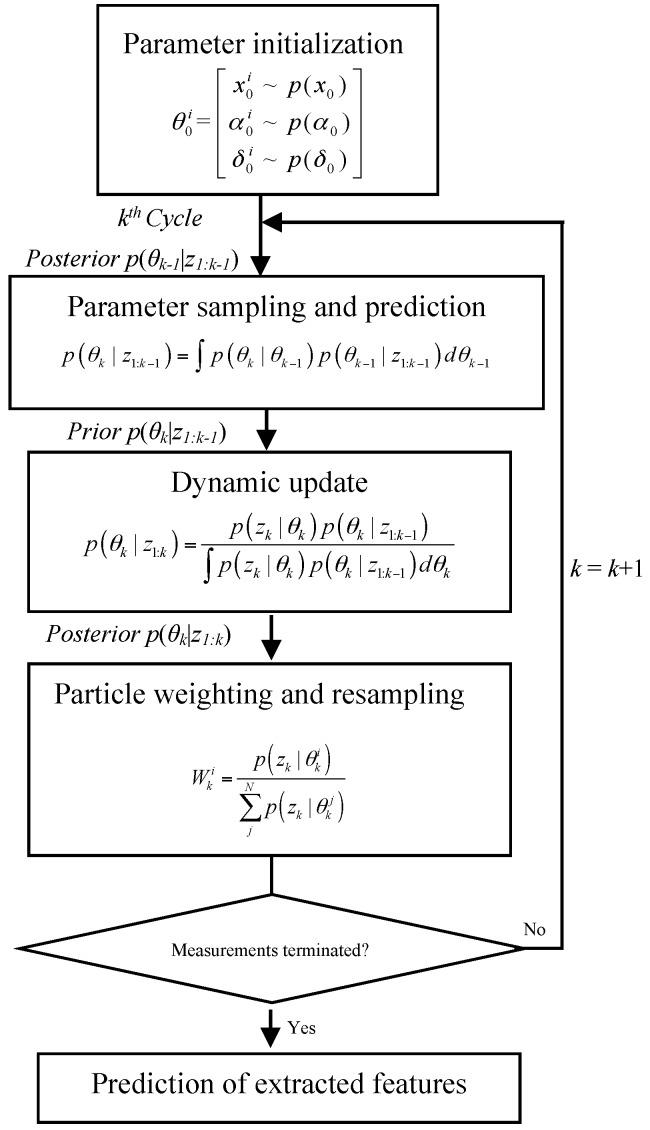
Particle filter (PF) prediction approach.

As illustrated in Figure 5, the recursive state estimation within the PF approach can be separated into five steps:

***Step 1: Parameter initialization***

The parameter vectors for both the state and measurement models can be expressed as *ʘ*(*x*, *α*, *δ*), and each parameter will be initialized by assuming a distribution drawn by the Monte Carlo simulation, with *N* particles.

***Step 2: Parameter sampling and prediction***

The prior probability density function (PDF) of the parameter vector at the *k*th cycle, *p*(*θ_k_*|*z*_1:*k*−1_), can be calculated based on the state model with the Chapman-Kolmogorov equation.

***Step 3: Dynamic updating***

With the new measurement, the posterior PDF at the *k*th cycle, *p*(*θ_k_*|*z*_1:*k*_), can be updated by using the Bayesian algorithm and the Markov assumption. The likelihood function of the *i*th particle at cycle *k*, *p*(*z_k_*|*θ_ik_*), can be expressed as a Gaussian distribution:

(10) $$p\left(z_{k}|\theta (x,b,\delta {)}_{k}^{i}\right)=\frac{1}{\sqrt{2\pi }\delta_{k}^{i}}\exp\left[-\frac{1}{2}{\left(\frac{z_{k}-x_{k}^{i}(b_{k}^{i})}{\delta_{k}^{i}}\right)}^{2}\right]$$

***Step 4: Particle weighting and resampling***

As calculated with Equation (11), the *i*th particle can be weighted with the particle weight as proportional to the PDF value of the likelihood function. To avoid the degeneracy problem in the iteration process, resampling based on the inverse cumulative density function method [29], was used to eliminate low-weight particles and condense high-weight particles.

(11) $$W_{k}^{i}=\frac{p\left(z_{k}|\theta_{k}^{i}\right)}{\sum_{j}^{N}p\left(z_{k}|\theta_{k}^{j}\right)}$$

***Step 5: Prediction of extracted features***

When the measurement is terminated at the *k*th step, the state finishes updating as *x_k_* ~ *p*(*x_k_*|*z*_1:*k*_) and the future states of the extracted features are predicted by extrapolating the estimated *k*th step state based on the state model.

## 4. Results and Discussion

In this section, the failure mechanism of the selected pc-WLED package under a predesigned accelerated degradation test was first identified by analyzing the extracted features from SPDs in Figure 2a. Next, the developed theory and methodology were validated with the collected SPDs to predict the color shift failure of the test sample.

### 4.1. Failure Mechanism Analysis

As introduced previously, SPD can be used to characterize the optical performances of pc-WLEDs. As a result, its deformation may indicate the specific failure mechanisms in an LED package, such as LED chip degradation, phosphor degradation, or polymer-based packaging materials (e.g., silicone lens and encapsulant) degradation. Furthermore, the failure mechanisms of a pc-WLED package were supposed to be related to the areas under the curve as shown in SPDs, *A*_B_ and *A*_Y_, which are dependent on the luminous energy emitted by the LED chip and phosphors, respectively.

**Figure 6:**
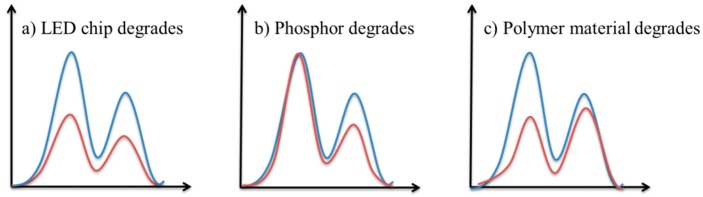
Failure mechanism classification in a pc-WLED package (the blue and red curves represent the initial and aged SPDs, respectively).

**Figure 7:**
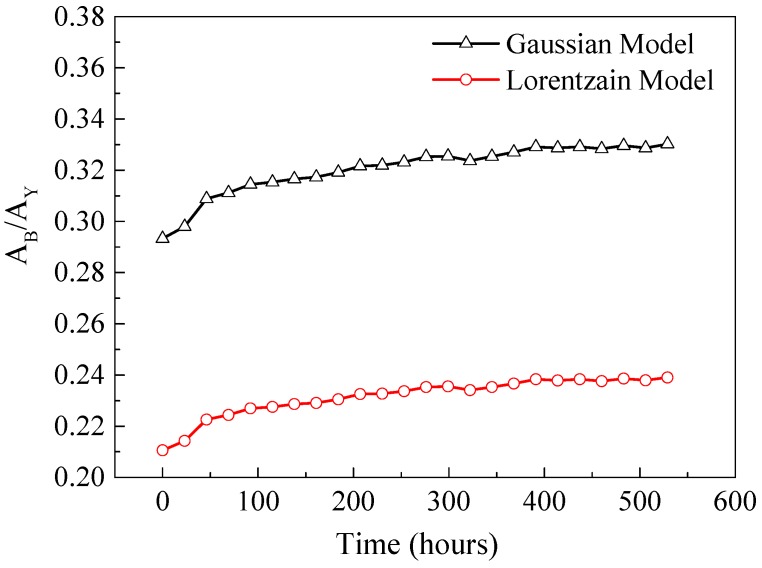
Ratio of extracted areas under the SPD curve.

As shown in Figure 6, three possible degradation scenarios of SPDs in a pc-WLED package are summarized as follows [30]: (1) If only the LED chip degrades, as the emission efficiency of phosphors depends on the energy of blue light emitted by LED chip, the areas of both the LED chip and phosphors are decreased proportionally (Figure 6a); (2) If only the phosphors degrade, the area under the long wavelength range is reduced more seriously (Figure 6b); (3) If only the polymer packaging materials degrades, the area of the LED chip in the SPD is decreased much more, because the polymer materials, such as silicone and epoxy, are always sensitive to short-wavelength light (Figure 6c). As shown in Figure 7, through the feature extraction by using two statistical models, the ratio of the areas under the curves of two ranges, *A*_B_/*A*_Y_, increases exponentially, which can support the conclusion that the phosphor degradation may be the dominant failure mechanism of the test sample under the designed accelerated degradation test.

**Figure 8:**
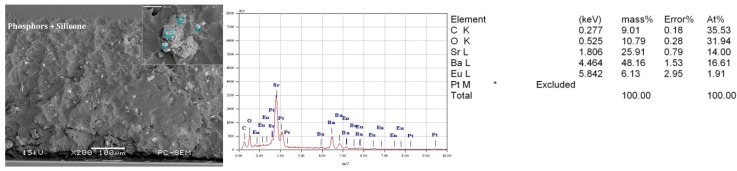
Chemical element analysis result of phosphors in the selected LED with the SEM-EDX.

**Figure 9:**
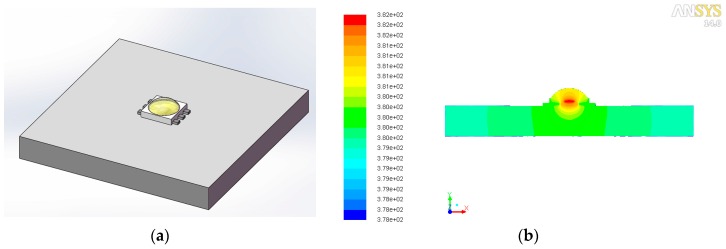
(**a**) The 3D model of test sample soldered on a substrate used for FEA simulation; (**b**) its simulated Kelvin temperature distribution.

Finally, to identify the degradation mechanisms of phosphors used in the selected test samples deeply, the chemical elements of the phosphor powders, those that were mixed in the silicone matrix, were analyzed with the SEM-EDX analyzer. From the results of the SEM-EDX image shown in Figure 8, it can be qualitatively determined that the used phosphors may be the europium ion doped strontium and barium silicate [31]. Considering the designed accelerated degradation test with the condition of the LED sample operated with *I*_c_ = 200 mA and *T*_a_ = 90 °C, its thermal distribution was simulated with the finite element analysis (FEA) method in the ANSYS FLUENT software and the material parameters used in FEA modeling are listed in Table 2. As shown in Figure 9, the highest temperature of the phosphor layer is more than 100 °C even without considering the self-heating effect from phosphors. According to the other studies on the thermal quenching effects of phosphors [32,33], the accelerated oxidization of europium ion caused by both the high-temperature heat treatment and blue light irradiation may result in the irreversible decrease in emission intensity of phosphors. That could be the main cause of the faster degradation of phosphors than that of other materials in the selected pc-WLED aged under this condition.

### 4.2. Color Shift Failure Prediction

**Figure 10:**
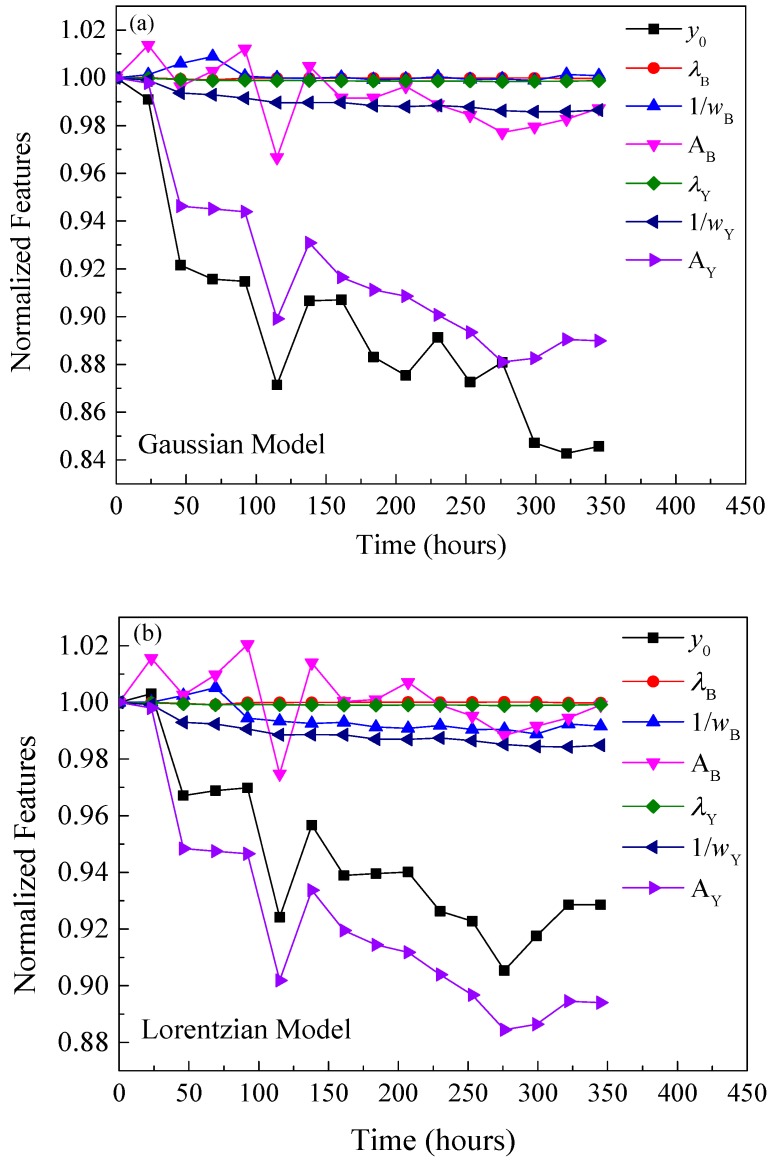
Shift trajectories of normalized features extracted from SPDs by (**a**) Gaussian model and (**b**) Lorentzian model until 345 h.

To validate the feasibility of the proposed color shift failure prediction method, this section used the extract features from collected SPDs until 345 h to predict the time to failure with the PF prediction method. As shown in Figure 10, three of the seven normalized features extracted by both statistical models, such as *λ*_B_, 1/*w*_B_, and *λ*_Y_, kept almost constant during the designed degradation test until 345 h. Thus, it is assumed that these three features are not degraded in this case, however, the remaining four features, including *y*_0_, *A*_B_, 1/*w*_Y_, and *A*_Y_, are supposed to exponentially degrade. Therefore, the state model described in Equation (8) can be rewritten as given in Equation (12), in which the shift trajectories of four normalized features are exponential modeled.

State model:

(12) $$x_{k}=\exp\left[-\alpha_{k}(t_{k}-t_{k-1})\right]x_{k-1};\;x_{k}=\left\{y_{0};A_{B};1/w_{B};A_{Y}\right\}$$

Table 3 shows the exponential curve-fitting results of the four normalized features collected until 345 h, which are used to estimate the initial distribution of state model parameters. *B*_G_ and *B*_L_ is the pre-parameters of state models from the Gaussian and Lorentzian model-fittings respectively. The initial distributions of the parameters defined in the vector of *ʘ_k_*(*x_k_*, *α_k_*, *δ_k_*) are assumed as uniform distributions, which can be represented in Equation (13). As there is an over-fitting for *A*_B_ with the Lorentzian model, it is assumed as the same uniform distribution from Gaussian model.

(13) $$\theta_{G0}^{i}=\left[\begin{array}{c} x_{G0}^{i}\sim U\left(0.9,1.1\right)\\ \begin{array}{l} \alpha_{G0}^{i}|y_{G0}\sim U\left(4.0\times 10^{-4},5.0\times 10^{-4}\right)\\ \alpha_{G0}^{i}|A_{GB}\sim U\left(7.0\times 10^{-5},8.0\times 10^{-5}\right)\\ \alpha_{G0}^{i}|1/w_{GY}\sim U\left(3.0\times 10^{-5},4.0\times 10^{-5}\right)\\ \alpha_{G0}^{i}|A_{GY}\sim U\left(3.0\times 10^{-4},4.0\times 10^{-4}\right) \end{array}\\ \delta_{G0}^{i}\sim U\left(0.01,0.02\right) \end{array}\right]\;\theta_{L0}^{i}=\left[\begin{array}{c} x_{L0}^{i}\sim U\left(0.9,1.1\right)\\ \begin{array}{l} \alpha_{L0}^{i}|y_{L0}\sim U\left(2.0\times 10^{-4},3.0\times 10^{-4}\right)\\ \alpha_{L0}^{i}|A_{LB}\sim U\left(7.0\times 10^{-5},8.0\times 10^{-5}\right)\\ \alpha_{L0}^{i}|1/w_{LY}\sim U\left(4.0\times 10^{-5},5.0\times 10^{-5}\right)\\ \alpha_{L0}^{i}|A_{LY}\sim U\left(3.0\times 10^{-4},4.0\times 10^{-4}\right) \end{array}\\ \delta_{L0}^{i}\sim U\left(0.01,0.02\right) \end{array}\right]$$

**Figure 11:**
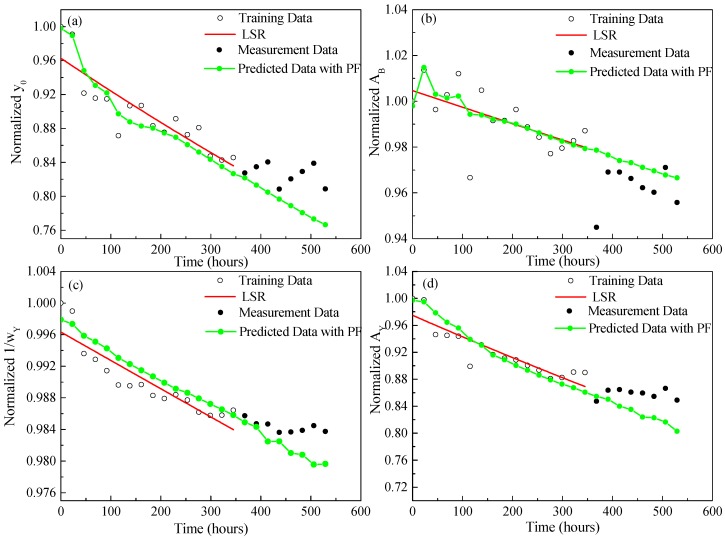
PF prediction results of four normalized features extracted from the Gaussian model until 529 h (**a**) Normalized *y*_0_; (**b**) Normalized *A*_B_; (**c**) Normalized 1/*w*_Y_; (**d**) Normalized *A*_Y_.

**Figure 12:**
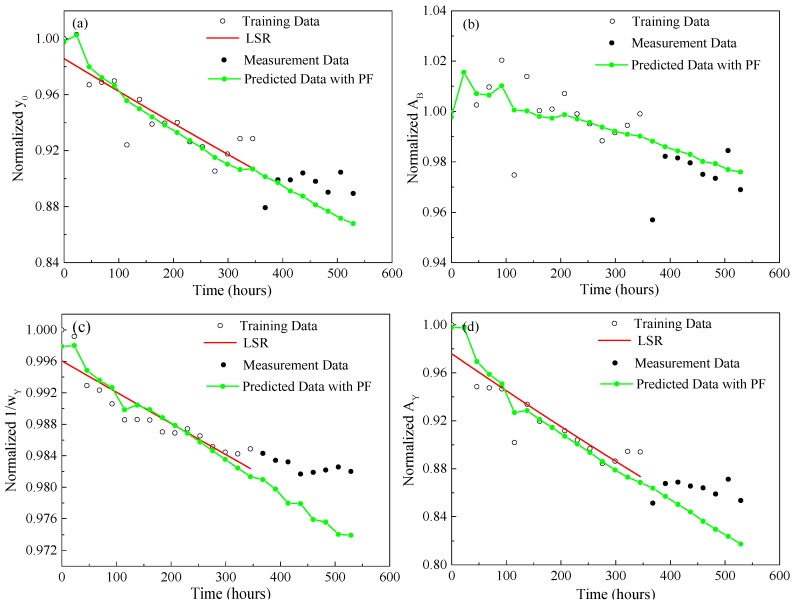
PF prediction results of four normalized features extracted from the Lorentzian model until 529 h (**a**) Normalized *y*_0_; (**b**) Normalized *A*_B_; (**c**) Normalized 1/*w*_Y_; (**d**) Normalized *A*_Y_.

Figure 11 and Figure 12 show the prediction results of four normalized features extracted from both Gaussian and Lorentzian models until 529 h, which indicate that the median values of the four normalized features predicted by the PF approach are relatively close to the actual measurement data marked with black dots. Meanwhile, as it estimates and updates the parameter vector dynamically by absorbing new measurements with considering the measurement noise, the PF approach takes the measurement dynamics and uncertainties into account in predicting the state model parameters.

**Figure 13:**
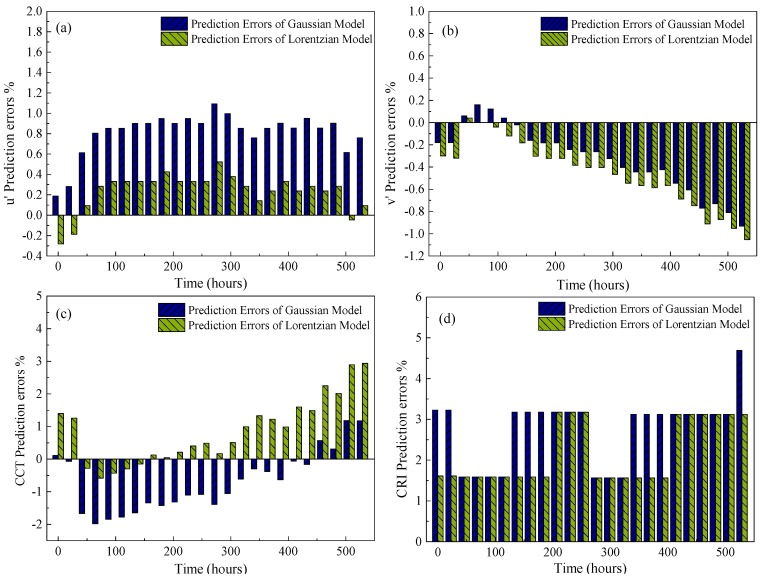
Prediction errors of (**a**) *u*′; (**b**) *v*′, (**c**) correlated color temperature (CCT) and (**d**) color rendering index (CRI) based on the Gaussian and Lorentzian models.

Next, based on the predicted features, the future SPDs of the test sample after 345 h can be predicted by reconstructing the Gaussian and Lorentzian models as shown in Equations (6) and (7). Then, the color coordinates (*u*′,*v*′) in the CIE1976 color space, the CCTs and CRIs can be inferred according to the predicted SPDs. The error percentages (Equation (14)) between predicted values and real measurements are calculated and the results are shown in Figure 13, which reveals that: (1) the absolute prediction errors of *u*′ and *v*′ based on the two statistical models can be controlled under 1% with the proposed PF approach, in which the approach with the Gaussian model has the better prediction accuracy in *u*′ and that with the Lorentzian model has less prediction error in *v*′; (2) the prediction errors of both the CCTs and CRIs can be controlled under 5%.

(14) $$Error\%=\frac{\left[X_{\mathrm{Predicted}}-X_{\mathrm{Real}}\right]}{X_{\mathrm{Real}}}\times 100\%$$

where *X* represents that *u*′,*v*′, CCT and CRI.

**Figure 14:**
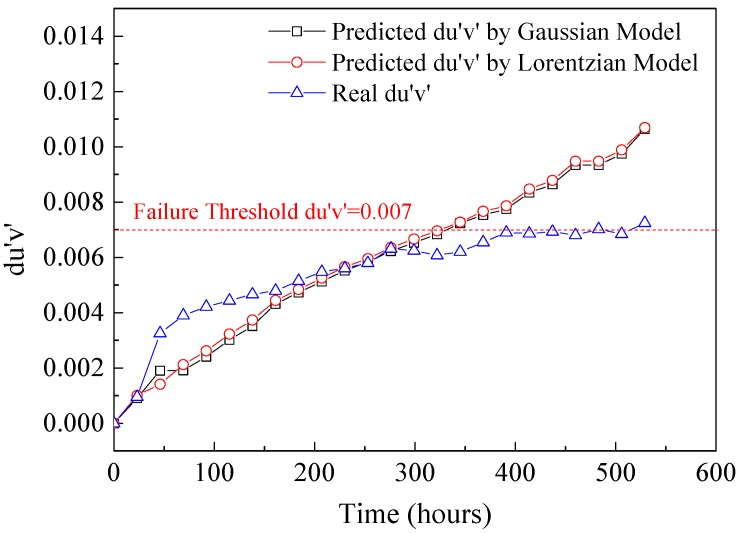
Prediction results of *du*′*v*′ based on the Gaussian and Lorentzian models.

Finally, the Euclidean distance between the original color coordinates and shifted coordinates in the CIE 1976 color space, *du*′*v*′, was calculated as shown in Equation (15) to represent the color shift of LEDs. Furthermore, the time when the predicted color coordinates shift to the failure thresholds defined as *du*′*v*′ = 0.007, can be estimated as the time to failure. The prediction result of *du*′*v*′ is shown in Figure 14, and it can be concluded that the predicted times to color shift failure from both statistical models are similar and they are earlier than the actual failure time. It could be a positive alarm to the test sample under the reliability testing.

(15) $$du\prime v\prime =\sqrt{{\left(u\prime -u\prime_{0}\right)}^{2}+{\left(v\prime -v\prime_{0}\right)}^{2}}$$

## 5. Conclusions

Traditionally, most of the concerns for the reliability of LEDs have been focused on luminous flux depreciation rather than on color shift failure. However, with the expansion of LED applications, much attention has been paid to the color quality and color consistency. Because the SPD of a light source can be used as the quantitative inference of both its photometry and colorimetry performances, this paper predicted the color shift failure for a pc-WLED package under a degradation test by modeling the shift processes of features extracted from SPDs with a nonlinear filtering method. The results show that: (1) By analyzing the feature extracted from the SPDs with the Gaussian model, the phosphor degradation was identified as the dominant failure mechanism of test sample under the designed accelerated degradation test; (2) The proposed PF method, taking the measurement dynamics and uncertainties into state prediction, can achieve a dynamic and accurate prediction for the color coordinates (*u*′,*v*′) in the CIE1976 color space with prediction errors under 1% and the prediction errors of both the CCTs and CRIs can be controlled under 5%; (3) Meanwhile, the residual color lifetime of the selected LED can also be estimated by considering the predicted color coordinates shift to the defined failure thresholds. To continue this work, the proposed feature extraction method with statistical models and the color shift prediction method can be improved by considering their fundamental physical senses to solve the color qualification problems for high-color rendering LEDs with multiple phosphors, and the new LED packages with different packaging materials and structures.

## Figures and Tables

**Table 1:** Results of feature extraction and model selection.

Models	*y*_0_	*λ*_B_	*w*_B_	*A*_B_	*λ*_Y_	*w*_Y_	*A*_Y_	*R*^2^
Gaussian model	2.26 × 10^−5^	459.684	25.144	0.0249	573.775	82.259	0.0849	0.99175
Lorentzian model	–8.97 × 10^−5^	458.656	23.221	0.03089	574.349	96.326	0.1467	0.98638

**Table 2:** Material parameters used in fine element analysis (FEA) modeling.

Material Parameters	Air	LED Chip	Silicone	Lead and Thermal Pad	Substrate
Density (kg/m^3^)	1.225	6150	1200	8920	2700
Thermal conductivity (W/m·K)	0.0257	130	5	398	100
Specific heat (J/kg·K)	-	490	1700	390	880

**Table 3:** State model parameter estimation.

Models		*y*_0_	*A*_B_	*1/w*_Y_	*A*_Y_
Gaussian Model	***B_G_***	0.96267	1.00464	0.99632	0.97489
	***α_G_***	4.09 × 10^−4^	7.23 × 10^−5^	3.62 × 10^−5^	3.33 × 10^−4^
Lorentzian Model	***B_L_***	0.98579	0.07459	0.99607	0.97595
	***α_L_***	2.4 × 10^−4^	0.84435	4.0 × 10^−5^	3.21 × 10^−4^
